# Cognate Effects on Bilingual Lexical–Semantic Processing in Children: Insights from ERPs

**DOI:** 10.3390/bs16020294

**Published:** 2026-02-19

**Authors:** Chih Yeh, Kathrin Wicinski, Caroline F. Rowland, Sergio Miguel Pereira Soares

**Affiliations:** 1 Language Development Department, Max Planck Institute for Psycholinguistics, 6525 XD Nijmegen, The Netherlands; caroline.rowland@mpi.nl (C.F.R.); sergio-miguel.pereira-soares@mpi.nl (S.M.P.S.); 2 Max Planck School of Cognition, D-04103 Leipzig, Germany; 3 Faculty of Social Sciences, Radboud University, 6525 GD Nijmegen, The Netherlands; kathrin.wicinski@ru.nl; 4 Donders Institute for Brain, Cognition and Behaviour, Radboud University, 6525 HT Nijmegen, The Netherlands

**Keywords:** language development, lexical–semantic processing, cognate facilitation effect, event-related potentials, N400

## Abstract

This study investigates whether and, if so, how cognates facilitate lexical–semantic processing during early bilingual development. Additionally, we examine the interaction between the cognate facilitation effect (CFE) and bilingual experience factors, such as language proficiency, exposure, and age. We investigated language backgrounds and recorded event-related potentials during a semantic priming task in Dutch–German bilingual children. Most participants were Dutch-dominant, characterized by higher exposure and proficiency in Dutch. We compared the N400 response to target words preceded by semantically related cognate versus non-cognate primes. We found a reduced N400 effect (indexing cognate facilitation) only in the non-dominant language (nDL; German). Individual difference analyses further revealed that higher proficiency of nDL and increasing age attenuated the CFE. In contrast, higher cumulative exposure was associated with an amplified CFE. These findings suggest that cross-linguistic activation in lexical–semantic processing may benefit younger children with either lower proficiency or higher exposure to their non-dominant language during language processing. Together, the study offers direct neural evidence for bilingual cognate facilitation effects and highlights the importance of investigating interactions with external factors in early bilingualism. Future longitudinal research should examine whether cognate reliance serves as a temporary scaffolding mechanism for the acquisition of the non-dominant language.

## 1. Introduction

Cognates are translation equivalents across two languages that share meaning and overlap in phonological and/or orthographic form. Previous studies have suggested that cognates can benefit lexical development and processing in bilingual children. For example, multiple studies report that translation equivalents with form overlap tend to be learned more easily and earlier in children’s vocabularies (Bosch & Ramon-Casas, 2014; Bosma et al., 2019; Garcia-Castro et al., 2025; Goriot et al., 2021; Lindgren & Bohnacker, 2020; Mitchell et al., 2024). Experiments using picture naming, word recognition, lexical decision, and sentence-reading tasks find shorter reaction times and/or higher accuracy for cognates vs. non-cognates (Bosma & Nota, 2020; Duñabeitia et al., 2016; Iniesta et al., 2024; Koutamanis et al., 2024a; Poarch & van Hell, 2012; Schröter & Schroeder, 2016). These findings align with the cognate facilitation effect (CFE). Previous studies suggested that CFE may arise due to the co-activation of overlapping semantic and phonological/orthographical representations within an integrated bilingual lexicon. Such co-activation often leads to faster lexical–semantic access, the process of retrieving words’ meanings and forms from memory (see the Bilingual Interactive Activation Plus model, BIA+; Dijkstra & van Heuven, 2002).

### 1.1. The Role of Age and Bilingual Experience

Some studies also addressed how cognates interact with bilingual language development by comparing CFE across age groups. Specifically, these studies examine whether the processing advantage for cognates (e.g., recognizing *rose* faster because of the Dutch *roos*) changes as children mature. However, results have been inconsistent. For instance, two studies report that sensitivity to cognates increases with age. Bosma et al. (2019) and Goriot et al. (2021) found that in younger children (ages 5–8 and ages 4–12), the CFE tends to emerge or increase with age, which they attribute to growing linguistic awareness and greater sensitivity to cross-linguistic phonological regularities. In contrast, Duñabeitia et al. (2016) reported that in older children and adolescents (ages 8–15), the CFE decreases with age. They suggest that increased reading experience and maturing language control mechanisms may strengthen cross-language inhibition and thus reduce CFE over time.

Several of the studies reviewed above have also investigated the role of individual bilingual experiences on the cognate status. Overall, they found asymmetric CFE in the two languages, where language exposure and proficiency appeared to modulate the effect (Bosma et al., 2019; Bosma & Nota, 2020; Duñabeitia et al., 2016; Goriot et al., 2021; Koutamanis et al., 2024a). These studies typically categorize participants’ two languages as dominant language (DL) and non-dominant language (nDL) based on exposure and/or objective proficiency measures. Several developmental studies report CFEs when targets are in the nDL (Bosma et al., 2019; Bosma & Nota, 2020; Koutamanis et al., 2024a). These findings align with the BIA+ model, which predicts faster activation of words in the DL and a substantial influence of DL activation on translation equivalents in the weaker language. Bosma and Nota (2020) found shorter reading times for cognates only in the children’s nDL in a sentence-reading task. Koutamanis et al. (2024b) also observed a CFE in Dutch-dominant children in a lexical decision task with Dutch–Greek bilinguals. These results indicate that both age and bilingual experiences may shape early bilingual lexical–semantic development, and they underscore the need to measure and model bilingual experience factors, such as exposure, proficiency, and age, when investigating CFE in semantic processing.

### 1.2. Cognate Effects in Semantic Processing

Despite robust evidence for CFE at the level of lexical access in both lexical processing and early bilingual development, far less is known about how this effect extends beyond word recognition or lexical access to influence bilingual semantic processing during development. Only one behavioral study so far has directly compared the semantic processing of cognates and non-cognates, and few have systematically examined cognate status. Floccia et al. (2020) found no cognate advantage in an intermodal preferential-looking task, but their broad cognate definition and omission of language dominance measures may have obscured effects. Evidence from adult studies indicates that cognates can speed recognition and enhance downstream semantic prediction (Ito et al., 2025), and faster prime processing increases priming magnitude (Hutchison et al., 2008). These findings posit a plausible mechanism in which cross-language overlap accelerates lexical access to the prime. Such speeded processing, in turn, facilitates prediction and semantic integration, which is important during early bilingual development.

Other work, instead of comparing cognates and non-cognates, examined the role of language distance in semantic priming. These results provide an indirect lens on CFEs: semantic processing in closely related language pairs is likely to benefit more from cognates than processing in more distantly related pairs. Studies of closely related language pairs (24-month Spanish–English, De Anda & Friend, 2020; 30-month French–English, Jardak & Byers-Heinlein, 2019) report priming in both languages with no interaction with dominance. Similarly, an EEG study reported N400 priming in bilingual toddlers (24–30 months) and found more pronounced priming effects in French–Spanish than in French–English toddlers (Rämä et al., 2024). The researchers reported a higher proportion of the cognate words in the French–Spanish condition (42.5%) than French–English (27.5%), although they did not explicitly control that cognate status. Such differences may partly account for the stronger priming effect in the French–Spanish condition. By contrast, Singh (2014) found no cross-language priming when the prime was in the nDL for 30-month English–Mandarin bilinguals, suggesting that bilingual children may not have benefited from their DL because the large language distance between the DL and nDL limits cross-language support. These patterns are consistent with higher cognate frequency and greater lexical overlap in related languages (Schepens et al., 2013), which would enhance cross-language activation and support semantic processing in the nDL. Together, these language distance findings provide converging, indirect evidence, implying that cognates may facilitate semantic processing by accelerating prime recognition and strengthening cross-language links.

### 1.3. The Present Study

The preceding review of behavioral and neural data tentatively suggests that cognates influence semantic processing; however, a systematic examination of CFE on semantic processing and development is currently lacking. To address this gap, the present study utilizes an auditory semantic priming paradigm alongside electroencephalogram (EEG) recordings in Dutch–German bilinguals (a closely related language pair; Schepens et al., 2013). We targeted the 2-to-6-year age range to capture the developmental trajectory of the CFE during the most dynamic period of lexical acquisition (DeAnda et al., 2016). Covering this broad window allows us to examine how sensitivity to cognates evolves from early word learning through the preschool years, providing a more granular view of how bilingual experience impacts CFE and semantic processing over time. Here, we use the N400, a well-established event-related potential (ERP) index of lexical–semantic retrieval (Delogu et al., 2019), as the primary neural signature of processing. The N400 is typically larger (more negative amplitude) for semantically incongruent than for congruent word pairs and is interpreted as reflecting increased semantic integration demands (Kutas & Federmeier, 2011). In priming paradigms, reduced N400 amplitude (i.e., less negative) for primed targets is taken as evidence of facilitated semantic access and activation of the semantic network. These features provide an online, response-independent index of cognitive processing and allow us to capture neural effects comparable to previous behavioral findings (e.g., Bice & Kroll, 2015).

With this in mind, the present study aims to (1) provide a neural-level test of CFE and (2) systematically investigate the moderating role of bilingual experience variables (specifically, language exposure, proficiency, and age). Our primary hypothesis is that cognate primes will facilitate the semantic processing of target words. We tested this by comparing the N400 responses of Dutch–German bilingual children listening to word pairs containing cognate primes (e.g., Baum/boom; “tree” in German and Dutch) versus non-cognate primes (e.g., Teller/bord; “plate” in German and Dutch). Given that a reduction in N400 amplitude indexes easier semantic integration, we specifically predicted smaller N400s for trials involving cognate primes. Furthermore, based on prior work, we predicted a stronger CFE in the children’s non-dominant language. Given the mixed developmental findings, we made no directional prediction for the effect of age.

## 2. Materials and Methods

### 2.1. Participants

Forty typically developing Dutch–German bilingual children aged between 2 and 6 years (mean = 3.99, SD = 1.07, range = 2.08–5.75, 15 females) participated in the study. We recruited these participants through the Baby and Child Research Centre in Nijmegen, The Netherlands. During the recruitment phase, we ensured that participants met specific inclusion criteria. Specifically, we verified that all children had normal hearing and vision, no diagnosed developmental disorders, and no significant exposure to languages other than Dutch or German. Furthermore, we confirmed that every participant received at least 10% of their language input in each of the two languages (Hoff et al., 2012; Hoff & Ribot, 2017). Geographically, most families resided in the Dutch–German border region; specifically, 37 families lived in the Netherlands, while three families lived in neighboring Germany. Caregivers reported a predominantly high socioeconomic status (SES) background. Regarding household income, 90% of families reported earning more than €3000 per month (with 75% exceeding €3800). Similarly, parental education levels were high, with 79.5% of caregivers holding a university degree. The study received approval from the Ethical Board of Social Sciences at Radboud University in Nijmegen, The Netherlands. Prior to the first experimental session, we provided caregivers with detailed information regarding the study procedures and obtained their written informed consent. Families received either €50 or €40 plus a children’s book as compensation.

### 2.2. Language Background Assessment

#### 2.2.1. Cross-Linguistic Lexical Task (CLT)

We assessed children’s receptive vocabulary using the Cross-linguistic Lexical Task (CLT; Haman et al., 2015), part of the LITMUS test battery (https://www.bi-sli.org/crosslinguistic-lexical-task, accessed on 11 February 2026). The CLT was developed for bilingual populations between 3 and 7 years of age and is available in multiple languages, making it suitable for cross-linguistic comparisons (analyses across different language systems) and within-child comparisons of lexical knowledge (the breadth and depth of a child’s vocabulary). We selected the CLT because it is specifically designed for bilingual populations; the test items were carefully selected based on Age of Acquisition (AoA) and complexity to ensure cross-linguistic equivalence and minimize cultural bias. In this study, we administered the comprehension component of the Dutch (CLT-NL; Van Wonderen & Unsworth, 2021) and the German version (CLT-DE; Rinker & Gagarina, 2017). The comprehension task follows a four-picture choice format: after viewing four images, the child hears an audio prompt and selects the picture that matches the target noun or verb. Each domain (nouns, verbs) includes 32 test items. Accuracy in the CLT reflects the child’s receptive vocabulary size and provides a reliable estimate of lexical knowledge in each language, independent of production abilities. The task’s standardized design across languages enables direct comparison of lexical development across two languages. In this study, the CLT was administered in Dutch and German, respectively, following the two EEG recording sessions (see Section 2.3.2 Procedure).

#### 2.2.2. Quantifying Bilingual EXperience (Q-BEx)

We collected children’s bilingual experience using the Q-BEx parental questionnaire (De Cat et al., 2023), which quantifies a bilingual child’s language background and experience. We chose this tool because it captures the dynamic nature of bilingualism by estimating cumulative exposure rather than current input alone, providing a more reliable metric of the nature of the linguistic input that shapes lexical development over time. Participants’ caregivers completed the questionnaire, which included modules on background information (e.g., age, family background), risk factors (e.g., language developmental delays), language exposure and use, the richness of linguistic experience, and language mixing. For the present study, we focused solely on the language exposure and use module, using the resulting cumulative estimates as an index of language exposure. To calculate cumulative exposure, caregivers first identified significant time points where a child’s language exposure changed. These points defined distinct time periods, and for each period, the caregiver reported the percentage of exposure to each language. Then, we calculated the total cumulative exposure for each language by multiplying the duration of each period (in months) by the percentage of exposure to that language and summing these values across all periods. The percentage of cumulative language exposure was then determined by dividing this total cumulative exposure score for a language by the child’s current age in months. In the current study, the Q-BEx was completed online by the caregiver(s) before the experiment and took approximately 15 to 30 min to finish.

### 2.3. Semantic Priming Paradigm

#### 2.3.1. Materials

We have pre-registered this study (Open-Ended Registration; Wicinski et al., 2025) on OSF (Open Science Framework). The materials were a subset from a larger study investigating lexical–semantic development in Dutch–German bilingual children using within- and across-language semantic priming with noun and verb pairs^1^ (for more details, see footnote 1 and the preregistration, Yeh et al., 2025). The current study focuses specifically on the CFE on semantic processing, given the N400’s sensitivity to various semantic factors (e.g., word frequency, concreteness, and semantic anomalies; see Kutas & Federmeier, 2011, for a review). To ensure that any reduction in the N400 response is solely due to cognate status, here, we only compare semantic-related noun pairs within each language.

The stimuli comprised 124 nouns in Dutch and German, arranged into 62 prime-target word pairs (32 Dutch and 31 German). To ensure that the words were familiar to the children, we selected the words from the Dutch and German McArthur Communicative Development Inventory (CDI-WS) (Szagun et al., 2023; Zink & Lejaegere, 2003), with a few additional German words sourced from a Goethe Institute A1-level word list (Goethe-Institut, 2004). To ensure uniform acoustic quality and native pronunciation, stimuli were recorded in a soundproof booth by two female speakers per language (Dutch and German). All audio files were subsequently processed using the *pydub* library in Python (version 3.8) to normalize intensity (RMS amplitude) (Robert, 2018).

Cognate status was determined by four native Dutch–German bilingual adults. Based on their ratings, there were 16 German cognates in the Dutch condition and 19 Dutch cognates in the German condition. We also calculated the normalized Levenshtein Distance (NLD), a measure of phonetic similarity for determining the degree of phonological overlap between the translation equivalents (Dijkstra et al., 2010; Schepens et al., 2013; Tokowicz et al., 2002), and included word frequency (SUBTLEX; Brysbaert et al., 2011; Keuleers et al., 2010) and word length as control variables. Details are presented in Table 1. These measures confirmed the rating results: cognate conditions had significantly higher phonetic similarity than non-cognate conditions (Dutch: t(30) = 6.88, *p* < 001; German: t(29) = 7.74, *p* < 001). Crucially, we found no significant differences between the cognate and non-cognate word pairs for word length (Primes: Dutch t(30) = −1.01, *p* = 0.32; German t(29) = −1.43, *p* = 0.16; Targets: Dutch t(30) = −0.47, *p* = 0.64; German t(29) = 0.73, *p* = 0.47), word frequency (Primes: Dutch t(29) = 1.91, *p* = 0.07; German t(28) = 0.36, *p* = 0.72; Targets: Dutch t(28) = 0.89, *p* = 0.38; German t(28) = 0.15, *p* = 0.90), or semantic relatedness between the pairs (Dutch t(30)= −0.35, *p* = 0.73; German t(29) = 0.74, *p* = 0.47). Thus, our two conditions varied primarily in their phonetic overlap. A detailed overview of the stimuli and their characteristics is provided in Appendix A Table A1.

#### 2.3.2. Procedure

The study was conducted across two sessions. Each of the two visits comprised an EEG recording and a CLT session. The order of languages of CLT (Dutch vs. German) was counterbalanced across the two visits. Additionally, within each CLT assessment, the administration order of the subsections (nouns vs. verbs) was randomized and counterbalanced. During the EEG recording, the participant and their parents were seated in a soundproof, electrically shielded booth. Stimuli were presented via two speakers at 65 dB. In each trial, a cognate or non-cognate prime was presented within a carrier phrase within the language (examples for Dutch: cognate prime: “*een sok*”, a sock; non-cognate prime: “*een winkel*”, a store), followed by a semantically related target word (e.g., “*schoen*”, shoe; “*bakker*”, bakery). The interstimulus interval (ISI) between the prime phrase and target word was 500 ms; the intertrial interval (ITI) was 2200 ms.

To keep the participants engaged and calm, silent cartoon videos unrelated to the stimuli were shown on a screen; quiet fidget toys and coloring materials were also provided during the experiment. Importantly, because participants process the auditory stimuli passively, developmental EEG studies often prioritize the acquisition of high-quality neural data over enforcing attentional focus (Hervé et al., 2022). To prevent inadvertent cueing by the parent, parents wore headphones delivering masking audio (music and speech). After each EEG recording, participants completed the CLT with an experimenter (see Section 2.2.1).

#### 2.3.3. EEG Recording and Analysis

The electroencephalogram (EEG) was continuously recorded using 32 active Ag/AgCl electrodes (actiCAP) (FP1, FP2, F7, F3, Fz, F4, F8, FT9, FC5, FC1, FC2, FC6, FT10, T7, C3, Cz, C4, T8, TP9, CP5, CP1, CP2, CP6, TP10, P7, P3, Pz, P4, P8, O1, Oz, O2) from the 10–20 system (BrainAmp DC amplifier, and Brain Vision Recorder Brain Products GmbH, Gilching, Germany). All electrodes were online referenced to FCz. The O1 and O2 electrodes were placed on the mastoids as offline reference electrodes (“TP9L”, “TP10L”). This different configuration provided offline re-referencing without compromising relevant data acquisition, as occipital channels are not critical for the analyses conducted in this paradigm. The two frontal electrodes above the eyes (Fp1, Fp2) were used to capture vertical eye movements, and the two electrodes at the outer canthi of the eyes (FT9, FT10) were used to capture horizontal eye movements. Electrode impedance levels were generally kept below 25 kΩ. Data were recorded at a sampling rate of 500 Hz, with an online time cut-off of 10 s and a high cut-off of 1000 Hz.

For the ERP analysis, continuous data were pre-processed in MATLAB (R2024a) using EEGLAB (Delorme & Makeig, 2004) and ERPLAB (Lopez-Calderon & Luck, 2014). The continuous EEG was band-pass-filtered offline from 0.1 to 30 Hz. Epochs were created time-locked to the target word onset and spanned 1200 ms (−200 to 1000 ms relative to stimulus onset). Baseline correction was performed on the −200 to 0 ms pre-stimulus window. Automatic artefact detection removed bad channels and epochs with high-amplitude artefacts (±150 μV for EEG channels; ±250 μV for EOG channels). After removal of large artefacts, data were decomposed using independent component analysis (ICA); components reflecting eye movements were identified by component morphology and visual inspection and removed. On average, 1.63 eye movement components were removed per participant. A second round of manual artefact rejection was then performed to remove residual noise not captured by ICA. Missing channels were interpolated for 14 participants using spherical-spline interpolation (mean number of repaired channels = 1.7, range = 1–5). Artefact-free data were re-referenced to the average of the mastoid electrodes (TP9L, TP10L). Only participants with at least eight artefact-free trials per condition were included in the final analysis. Overall, 20.84% of trials were rejected on average. Eleven participants were rejected.

As proposed in the preregistration, we firstly performed cluster-based permutation analyses (CBPA; Maris & Oostenveld, 2007) in Fieldtrip (Oostenveld et al., 2011) to assess differences between the cognate and non-cognate. In the expected N400 time window (250–550 ms), the test revealed no significant differences in either language. However, an exploratory open-window search (0–1000 ms) identified a significant negative cluster in the German condition (*p* = 0.02) from 726 to 998 ms over fronto-central electrodes (21 channels). Within this cluster, non-cognates displayed more negative amplitudes than cognates. While we initially expected the cognate effect to manifest as a reduced N400 (Kutas & Federmeier, 2011), this later time window aligns with distinct neural signatures observed in bilingual children’s non-dominant language. For instance, Sirri and Rämä (2019) found a late anterior negativity in French–Spanish bilingual children (aged 2–4), which they attributed to less efficient semantic processing. Similarly, Conboy and Mills (2006) linked late negativity to increased attentional demands in toddler word processing. Thus, the late negativity observed here may reflect delayed semantic integration in the non-dominant language, with the reduced amplitude for cognate targets indicating a reduction in these processing demands.

Despite this exploratory finding, our primary hypothesis specifically concerned the earlier N400 component. Cluster-based tests, while robust for exploratory searches, can lack sensitivity for specific effects if the signal is spatially broad or temporally fleeting (Sassenhagen & Draschkow, 2019). Therefore, to rigorously test our a priori N400 predictions, we proceeded to a region-of-interest (ROI) analysis on the mean amplitudes within the 250–550 ms window.

While our preregistration specified a Bayesian framework, we opted for a frequentist linear mixed-effects (LME) model using the lme4 package (Bates et al., 2015) in R. This adjustment was made to facilitate standard significance testing and ensure comparability with the existing frequentist literature in this domain. The final model included fixed effects for cognate status (levels: cognate, non-cognate; reference: cognate), language (levels: Dutch, German; reference: German), and topographic distribution (levels: frontal–central, posterior; reference: frontal–central), along with their full interactions. To account for individual variability in processing, the model incorporated random intercepts for subjects and random slopes for cognate status by subject, which allowed the effect of cognate status to vary across individual participants.

#### 2.3.4. Individual Differences Analysis

The effects of language exposure, proficiency, and age on the cognate facilitation effect (CFE) were analyzed using linear mixed-effects models. For each participant, N400 mean amplitudes were computed for every condition at each channel. Language exposure was operationalized as the percentage of German exposure reported in the Q-BEx questionnaire, since Dutch and German together accounted for 100% of cumulative exposure; thus, higher German exposure values corresponded to lower Dutch exposure. Proficiency was indexed by overall accuracy in Dutch or German, depending on the language dominance (which would be determined by the amount of language exposure). The initial model specification included fixed effects of cognate status, age, and percentage of German exposure, as well as interactions between cognate status and each of these predictors. Random intercepts for participants were also included. To address potential collinearity, we first assessed intercorrelations among predictors. Based on these results, highly correlated predictors were included in separate models to ensure stability.

## 3. Results

This study incorporates both behavioral and neural measures, each with different sample sizes, in order to maximize participant inclusion. For the ERP analyses, we report data from 29 participants (10 female; *M* = 4.16 years, *SD* = 1.07). Eleven participants were excluded from this dataset due to insufficient number of trials remaining after artifact removal (e.g., blinks, muscle noise and head movements). For the subsequent individual-level analyses, which combine ERP and behavioral data, further exclusions were necessary: two participants due to incomplete Q-BEx questionnaires (final *N* = 27) and one due to missing CLT task data (final *N* = 28). As a result, the analyses in Section 3.1 and Section 3.3 rely on different sample sizes. We included 27 participants in the language exposure and age model, but 26 participants in the language exposure and proficiency model. This variation reflects the availability of data for each specific predictor.

In summary, the results confirmed that the majority of participants were Dutch-dominant based on language background measures. Neural data indicated a CFE, indexed by reduced N400 effect, only in the non-dominant language (German). Finally, linear mixed-effects modeling revealed that the effect was modulated by individual differences in age, language proficiency, and cumulative exposure, respectively. We detail these findings in the sections below.

### 3.1. Language Background

Descriptive results for the participant’s cumulative language exposure, as measured by the Q-BEx, and lexical proficiency in Dutch and German, assessed using the LITMUS Cross-linguistic Lexical Task (CLT), are presented in Table 2. Overall, both language background measures reveal a clear pattern of Dutch, which aligns with the demographic context that the majority of families reside in the Netherlands, where Dutch is the societal language. Consequently, German represents the non-dominant language for our sample on average. Based on the Q-BEx questionnaire (*N* = 27), twenty-two participants received more cumulative exposure to Dutch than to German, three were predominantly exposed to German, and two had balanced exposure. The CLT score (*N* = 28) also demonstrates an overall better proficiency in Dutch. Specifically, the accuracy for nouns is better than verbs regardless of language. No significant difference was observed in participants’ noun proficiency across languages (t(24) = 0.16, *p* = 0.88), whereas verb proficiency was significantly higher in Dutch (t(24) = 5.52, *p* < 0.001).

To examine the relationships between the behavioral measures, we conducted Pearson’s correlations including age, cumulative exposure to both languages, and CLT scores in both languages. The results shown in Table 3 revealed strong correlations between age and language proficiency (i.e., all the CLT scores).

### 3.2. ERPs

**Figure 1:**
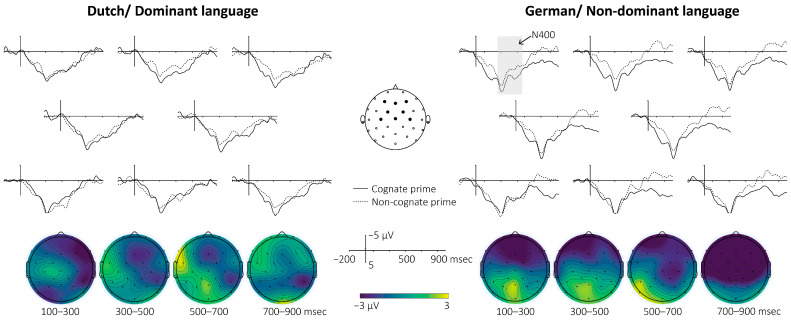
Grand-average event-related potentials (ERPs) of the N400 effects. The graphs present the ERPs recorded at representative electrode sites (F3, Fz, F4, FC1, FC2, C3, Cz, C4), indicated by the black dots on the head diagram. Negative voltage is plotted upward. The waveforms elicited by cognate prime (solid line) and non-cognate prime (dashed line) are overlaid. The grey-shadowed area indicates the time window of interest (250 to 500 ms). ERPs shown here were additionally filtered with a 15 Hz lowpass filter for visualization purposes. The topographic distribution demonstrates the difference wave between two conditions (non-cognate−cognate).

A linear mixed-effects model was used to investigate whether the N400 mean amplitude measured within the 250 to 550 ms time window differed between cognates and non-cognates in both Dutch and German. The mixed-effects model showed a marginal main effect of cognate status (model coefficient for non-cognate − cognate = −1.86, *p* = 0.048), which corresponded to cognates eliciting on average an effect that was 1.86 µV more positive than non-cognates (i.e., a smaller N400 response for cognates). In addition, the model revealed several interactions affecting N400 amplitude. The cognate × language interaction (β = 1.23, *p* = 0.048) indicated that the reduction in N400 for cognates versus non-cognates was reduced in Dutch. The cognate × topography interaction (β = 2.05, *p* < 0.001) showed that the N400 difference between conditions was smaller at posterior electrodes than at frontal–central electrodes. Language also interacted with topography (β = 1.71, *p* = 0.004), reflecting language-dependent differences in anterior–posterior amplitude. The three-way interaction between cognate status, language, and topography did not reach the significance threshold (β = −1.65, *p* = 0.053). However, the result suggested a trend where the reduction in the cognate effect in Dutch (compared to German) was stronger over posterior electrodes. Overall, cognates elicited a smaller N400 than non-cognates (≈1.9 µV), but this N400 difference is substantially modulated by both topographic distribution and language. Figure 1 displays the EEG waveforms. Table 4 details the fixed effects for the main ERP analysis of the semantic priming experiment. We report the subsequent analyses regarding how language proficiency and exposure modulate these effects in the ‘Individual Differences’ section (Section 3.3; see Table 5).

Post hoc planned pairwise comparisons on estimated marginal means (EMMs; emmeans; Lenth et al., 2025) with FDR correction revealed a significant cognate facilitation effect at frontal–central sites in German: cognate–non-cognate = 1.86 µV, SE = 0.94, 95% CI [0.02, 3.70], *p* = 0.0475 (FDR-adjusted), consistent with the cognate facilitation effect (smaller N400 for cognates), but not at frontal–central sites in Dutch (cognate–non-cognate = 0.63 µV, SE = 0.94, 95% CI [−1.21, 2.48], *p* = 0.50) or at posterior sites in either language (German: −0.19 µV, 95% CI [−2.00, 1.63], *p* = 0.84; Dutch: 0.23 µV, 95% CI [−1.58, 2.05], *p* = 0.80).

### 3.3. Individual Differences

The LME and post hoc analyses reported in Section 3.2 revealed significant differences in the German condition (frontal–central region) but not the Dutch condition. As such, here we only focus on the German conditions to examine how bilingual experience modulates the N400 cognate facilitation effect (CFE) in a non-dominant language. We tested whether the cognate effect was influenced by German proficiency, age, and cumulative exposure to German. Because age and proficiency were highly collinear (see Table 2), we fitted two separate models. In both models, we included interactions between cognate status and the individual predictors (e.g., cognate × age and cognate × exposure). However, we excluded the three-way interaction (e.g., cognate × age × exposure) and the interaction between predictors (age × exposure) after preliminary model checks revealed that including these terms introduced severe multicollinearity (correlations of fixed effects > 0.80), which compromised the stability of the estimates. Therefore, the final models were specified as follows: Model 1 included cognate status, German proficiency, cumulative exposure and their interaction while Model 2 included cognate status, age, cumulative exposure and their interaction.

The mixed-effects models revealed that cognate status was a robust predictor of N400 mean amplitude across both specifications (see Table 5). In Model 1, cognate words elicited significantly more negative amplitudes compared to non-cognates (β = −17.79, 95% CI [−21.77, −13.81]), and this effect was moderated by both German CLT accuracy and cumulative exposure to German. Specifically, higher CLT accuracy attenuated the cognate effect (β = 23.06, 95% CI [18.22, 27.91]), whereas greater cumulative exposure amplified it (β = −6.30, 95% CI [−10.33, −2.26]). In addition, German CLT accuracy alone was negatively associated with N400 amplitude (β = −18.62, 95% CI [−26.49, −10.74]), and cumulative exposure showed a modest positive association (β = 7.23, 95% CI [0.67, 13.79]).

Model 2, which included age as a predictor, confirmed the main effect of cognate status (β = −4.82, 95% CI [−8.03, −1.61]), while also revealing a significant negative effect of age (β = −1.75, 95% CI [−2.82, −0.69]). Age further interacted with cognate status, such that the cognate effect decreased with increasing age (β = 1.20, 95% CI [0.51, 1.89]). Cumulative exposure to German and its interaction with cognate status were not significant in this model.

Across both models, the cognate effect on N400 amplitude was robust, confirming that cognate words elicit enhanced neural responses compared to non-cognates in the non-dominant language. However, the factors moderating this effect differed depending on whether proficiency or age was considered as part of the model. In Model 1, higher German proficiency reduced the cognate facilitation effect, suggesting that more proficient bilinguals rely less on cross-linguistic form overlap. In contrast, greater cumulative exposure enhanced the effect, indicating that continued experience with the non-dominant language amplified sensitivity to cognate status. In Model 2, age emerged as a significant predictor, both directly influencing N400 amplitude and moderating the cognate effect, such that older participants showed a weaker facilitation effect.

## 4. Discussion

The current study directly investigated how the CFE influences online semantic processing in the developing brain, and the interaction between the CFE and individual bilingual experience variables. The results from the language background assessment and questionnaire indicated that the majority of our participants were Dutch-dominant, with German as their non-dominant language. The semantic priming experiment revealed a crucial finding: the CFE, indexed by a reduction in N400 amplitude in the cognate condition, was present in the nDL but absent in the DL. This suggests that bilingual speakers utilize cognate representations in the DL to support semantic processing in their nDL. Further analyses using linear mixed-effects (LME) models demonstrated a joint impact of age, language proficiency and cumulative language exposure on the N400 effect. Specifically, participants with lower nDL accuracy and higher nDL exposure showed a greater reliance on cognates during nDL processing. Finally, we discovered an age effect, which aligned with the proficiency findings, indicating that younger participants exhibited a stronger CFE during nDL processing.

This main finding confirmed that there was a CFE in semantic processing, but also raised a question regarding the underlying mechanism: how does a cognate prime, possessing a phonologically and semantically overlapping translation equivalent in the DL (e.g., Dutch oor), facilitate online semantic priming exclusively within the nDL (e.g., German Ohr–Mund)? The relationship between CFE and semantic processing is not as straightforward as in studies of word recognition and naming, where facilitation is typically observed in explicit measures (for example, shorter naming reaction times for cognates than for non-cognates). Semantic priming, in contrast, indexes more implicit and automatic activation. Below, we propose our interpretations of how cognates might influence semantic processing and development.

Cognate words are fundamentally acquired earlier and more easily than non-cognates in bilingual children’s vocabularies (Bosch & Ramon-Casas, 2014; Bosma et al., 2019; Garcia-Castro et al., 2025; Goriot et al., 2021; Lindgren & Bohnacker, 2020; Mitchell et al., 2024). That means bilingual children might be more familiar with cognate words than non-cognates. Garcia-Castro et al. (2025) suggested that the CFE on word acquisition is the result of enhanced cross-language co-activation: when a bilingual child is exposed to a word form, they activate the corresponding lexical representations of both languages. Since cognates receive stronger activation from their translation equivalent than non-cognates, children accumulate learning instances at a faster rate. As a result, the lexical representations of cognate words consolidate at earlier ages than those of non-cognate words.

Therefore, it is plausible that cognates facilitate semantic processing by accelerating recognition of the prime, which in turn enhances activation of the weaker lexical–semantic links in the nDL. This compensatory mechanism is relevant because lexical–semantic activation is typically slower in the nDL of bilinguals. For instance, Rämä et al. (2018) found that semantic priming effects in bilingual infants emerged only at longer Stimulus Onset Asynchronies (SOAs). Given that faster prime recognition has been shown to enhance semantic priming (Hutchison et al., 2008) and that previous studies indicate cognates accelerate word recognition (Duñabeitia et al., 2016; Koutamanis et al., 2024a, 2024b; Schröter & Schroeder, 2016), cognates may partially compensate for slower nDL activation. This supports more efficient semantic processing, as reflected in the reduced N400 amplitude for the cognate primes observed in our study.

Building on this interpretation, we speculate that in unbalanced bilingual children (biased to one of their native languages), cognate words with stronger lexical representations of their DL may serve as a bridge or scaffolding for that of their non-dominant languages’ development (Costa et al., 2005; Iniesta et al., 2024). This implies that as children accumulate concept and lexical representation mappings in their DL, the cognate link (the phonological overlap between translation equivalents) can strengthen the connections between the nDL representations and the shared concept. As a result, unbalanced bilingual children could build their nDL via their DL. This necessity for scaffolding is consistent with the “Weaker Links” Hypothesis (Gollan et al., 2008), which posits that the reduced exposure and use of the nDL lead to less frequent activation and, consequently, weaker lexical–semantic links within that language (Sirri & Rämä, 2019).

Our results revealing both a language proficiency-related and an age-related decrease in the CFE support this idea. These findings suggest that cognates not only facilitate online processing, but may also help unbalanced bilingual children establish stronger lexical representations of their nDL. The age-related decline in the CFE has also been reported in reading development, where increased reading experience and the maturation of language control mechanisms reduce cognate benefits (Duñabeitia et al., 2016). The developmental BIA model (BIA-d) (Grainger et al., 2010) accounts for this effect by proposing that form-based co-activation of translation equivalents decreases as proficiency increases, due to more effective inhibitory control mechanisms suppressing cross-linguistic interference. Duñabeitia et al. (2016) extend this view by emphasizing the role of developing attentional and control processes in attenuating the CFE during reading development. By extension, the consolidation of the lexical semantic system and improved language control mechanisms likely contribute to the age-related reduction in lexical semantic cognate facilitation observed in the present study.

Finally, we need to acknowledge several limitations of the present study. First, as we noted in Materials and Methods, our stimulus set was drawn from a subset of items from a larger study that was not originally designed to compare semantic priming effects between cognate and non-cognate primes. As a result, the present experiment included a relatively low number of trials, which precluded strict control over the cognate status of the target words. This matters because a greater proportion of trials with non-cognate primes preceding cognate targets may have attenuated the observed cognate effect by facilitating processing in the non-cognate condition. Indeed, across both languages, the proportion of cognate targets was higher in the non-cognate prime condition (Dutch: 69% vs. 50%; German: 75% vs. 55%).

Next, even though our final sample size (*N* = 27) is typical for developmental EEG research, it remains relatively modest given the complexity of the linear mixed-effects models we employed. The inclusion of multiple predictors and interaction terms increases the risk of overfitting and limits the statistical power to detect subtle effects. The modest nature of the N400 evidence (*p* = 0.048) reflects this issue; we detected this effect only in the hypothesis-driven ROI analysis and not in the more conservative cluster-based permutation test. This discrepancy highlights the trade-off between the sensitivity of hypothesis-driven ROI approaches and the strict error control of cluster-based permutation analyses, and thus these effects should be interpreted with caution. 

Despite the limitations regarding statistical power and sample size, these findings offer a theoretical basis for future research. If the CFE serves as a scaffolding mechanism for the non-dominant language, as our data tentatively suggest, this dynamic could eventually inform strategies in bilingual education. Consequently, future research should expand on these findings to better understand the developmental trajectory of the CFE. Beyond the necessity for larger, high-powered replications, longitudinal designs are essential to map how the reliance on cognates shifts over time within the same individuals. Such studies could determine whether the age-related reduction in CFE we observed represents a true developmental shift in lexical organization or simply a byproduct of increasing proficiency. Furthermore, to disentangle the role of phonological overlap from semantic overlap, future work should examine these effects in bilinguals acquiring language pairs with varying degrees of language distance (e.g., English–Dutch versus English–Spanish). Comparing these populations would clarify whether the scaffolding mechanism relies strictly on form similarity or if conceptual links alone are sufficient to drive cross-language activation in the developing brain.

In summary, our results provide tentative evidence for a CFE, suggesting that bilingual children growing up with two closely related languages can benefit from cognates during lexical–semantic processing in their non-dominant language. Consistent with the patterns observed in asymmetric activation, the magnitude of the CFE appears to be influenced by developmental stage, language proficiency, and cumulative exposure. These findings underscore the importance of considering both linguistic and developmental context in future studies of bilingual lexical–semantic processing.

## Figures and Tables

**Table 1:** Description of materials.

Condition	NLD (Phonetic)	Word Length (Phonemes)	Word Frequency (SUBTLEX)	Semantic Relatedness
German cognate prime (*N* = 19)				
Mean (SD)	0.89 (0.07)	4.11 (1.70)	60.13 (77.96)	4.70 (0.96)
Range	0.75–1.00	1–7	0.94–326.98	2.60–5.82
German non-cognate prime (*N* = 12)				
Mean (SD)	0.65 (0.10)	5.00 (1.71)	49.96 (66.95)	4.46 (0.80)
Range	0.51–0.8	3–9	2.36–204.34	2.18–5.70
Dutch cognate prime (*N* = 16)				
Mean (SD)	0.88 (0.09)	4.31 (1.96)	56.29 (78.98)	4.97 (0.91)
Range	0.70–1.00	2–9	1.69–247.48	2.70–6.00
Dutch non-cognate prime (*N* = 16)				
Mean (SD)	0.64 (0.11)	4.94 (1.53)	15.79 (22.92)	5.06 (0.67)
Range	0.42–0.78	3–8	0.5–67.28	3.30–5.91

**Table 2:** Descriptive statistics of language background measures (mean % score).

	Mean	SD	Range
Cumulative language exposure (*N* = 27)			
Dutch	66.37%	16.69%	16–88%
German	33.56%	16.68%	12–84%
Dutch CLT score (*N* = 28)			
Nouns	90.63%	20.31%	34.38–100%
Verbs	82.48%	20.84%	31.25–100%
German CLT score (*N* = 28)			
Nouns	90.51%	18.48%	46.88–100%
Verbs	66.85%	27.24%	9.38–93.75%

**Table 3:** Pearson’s correlations between behavioral measures (*N* = 26).

Variable	1	2	3	4	5	6	7	8	9	10	11	12	13	14	15
1. Age	1.00	--	--	--	--	--	--	--	--	--	--	--	--	--	--
2. Cumulated exposure of Dutch	−0.03	1.00	--	--	--	--	--	--	--	--	--	--	--	--	--
3. Cumulated exposure of German	0.03	−1.00 ***	1.00	--	--	--	--	--	--	--	--	--	--	--	--
4. Cumulated use of Dutch	−0.19	0.84 ***	−0.84 ***	1.00	--	--	--	--	--	--	--	--	--	--	--
5. Cumulated use of German	0.20	−0.83 ***	0.84 ***	−1.00 ***	1.00	--	--	--	--	--	--	--	--	--	--
6. Dutch noun accuracy	0.59 **	−0.24	0.24	−0.15	0.15	1.00	--	--	--	--	--	--	--	--	--
7. Dutch verb accuracy	0.75 ***	0.12	−0.12	0.11	−0.10	0.81 ***	1.00	--	--	--	--	--	--	--	--
8. German noun accuracy	0.64 ***	−0.24	0.25	−0.31	0.32	0.60 **	0.61 **	1.00	--	--	--	--	--	--	--
9. German verb accuracy	0.83 ***	−0.14	0.14	−0.20	0.21	0.76 ***	0.87 ***	0.78 ***	1.00	--	--	--	--	--	--
10. Dutch accuracy	0.71 ***	−0.04	0.04	−0.01	0.01	0.94 ***	0.96 ***	0.64 ***	0.86 ***	1.00	--	--	--	--	--
11. German accuracy	0.81 ***	−0.18	0.18	−0.25	0.26	0.75 ***	0.83 ***	0.89 ***	0.98 ***	0.83 ***	1.00	--	--	--	--
12. All accuracy	0.80 ***	−0.12	0.12	−0.14	0.14	0.88 ***	0.93 ***	0.80 ***	0.96 ***	0.96 ***	0.96 ***	1.00	--	--	--
13. Relative accuracy	−0.22	0.24	−0.25	0.41 *	−0.43 *	0.28	0.16	−0.49 *	−0.27	0.23	−0.35	−0.07	1.00	--	--
14. Relative noun accuracy	0.18	−0.09	0.08	0.08	−0.10	0.72 ***	0.48 *	−0.12	0.27	0.62 ***	0.15	0.40 *	0.78 ***	1.00	--
15. Relative verb accuracy	−0.52 **	0.46 *	−0.46 *	0.56 **	−0.57 **	−0.30	−0.24	−0.65 ***	−0.68 ***	−0.28	−0.71 ***	−0.52 **	0.77 ***	0.20	1.00

(Note: the reported correlation coefficients are based on a reduced sample size (*N* = 26) due to the exclusion of participants with missing data for both language exposure and proficiency measures. Please also note that the presented *p*-values have not been corrected for multiple comparisons. The significance legend used throughout the results is *** <0.001; ** <0.01; * <0.05; <0.1).

**Table 4:** Results of linear mixed-effects model.

Formula: N400~Cognate Status × Language × Topographic Distribution + (1 + Cognate|Subject)			
Predictors	Estimates	CI	*p*
(Intercept)	7.85	6.56–9.14	<0.001
Cognate status	−1.86	−3.70–−0.02	0.048
Language	−0.34	−1.20–0.52	0.442
Topographic distribution	−5.55	−6.39–−4.72	<0.001
Cognate status × Language	1.23	0.01–2.45	0.048
Cognate status × Topographic distribution	2.05	0.87–3.23	0.001
Language × Topographic distribution	1.71	0.53–2.89	0.004
Cognate status × Language × Topographic distribution	−1.65	−3.31–0.02	0.053
Random effects			
σ2	39.20		
τ00 _Subject_	9.77		
τ11 _Subject.Cognate_	20.00		
ρ01 _Subject_	−0.60		
ICC	0.23		
N _Subject_	29		
Observations	3480		
Marginal R^2^/Conditional R^2^	0.083/0.290		

**Table 5:** Results of two mixed-effects models. The formula for Model 1 is N400~Cognate × (German CLT Accuracy + Cumulated exposure of German) + (1 | Subject); for Model 2, it is N400~Cognate × (Age + Cumulated exposure of German) + (1 | Subject).

	N400 Mean Amplitude (µV)	
Predictors	Model 1 Estimate (CI)	Model 2 Estimate (CI)
(Intercept)	18.04 *** (11.58–24.51)	10.81 *** (5.84–15.78)
Cognate status	−17.79 *** (−21.77–−13.81)	−4.82 ** (−8.03–−1.61)
German CLT accuracy	−18.62 *** (−26.49–−10.74)	-
Age	-	−1.75 ** (−2.82–−0.69)
Cumulated exposure of German	7.23 * (0.67–13.79)	4.87 (−1.41–11.14)
Cognate status × German CLT accuracy	23.06 *** (18.22–27.91)	-
Cognate status × Cumulated exposure of German	−6.30 ** (−10.33–−2.26)	−3.17 (−7.23–0.88)
Cognate × Age	-	1.20 ** (0.51–1.89)
Random effects		
σ2	48.89	51.15
τ00 _Subject_	6.98	6.45
ICC	0.12	0.11
N _Subject_	26	27
Observations	1560	1620
Marginal R^2^/Conditional R^2^	0.074/0.190	0.038/0.146
AIC	10,569.7	11,045.5
BIC	10,612.5	11,088.7

(Note: *** <0.001; ** <0.01; * <0.05; <0.1).

## Data Availability

The data presented in this study are available on request from the corresponding author. The data are not publicly available due to privacy or ethical restrictions.

## Note

1 Yeh et al. (2025) utilied a semantic priming paradigm to investigate bilingual lexical-semantic processing. The experimental design included both within-language (e.g., Dutch prime followed by Dutch target) and across-language (e.g., Dutch prime followed by German target) word pairs. The full factorial design crossed four independent variables: language context (within- or across-language), prime language (Dutch or German), word category (noun or verb), and semantic relatedness (related or unrelated). This resulted in a total of 16 experimental conditions.

## Appendix A

**Table A1:** Stimulus list with normalized phonetic Levenshtein Distance, word length (phonemes), and SUBLEX word frequency.

**Dutch** **Prime**	**German Translation**	**Cognate Status**	**Prime NLD**	**Word Frequency**	**Word Length**	**Dutch** **Target**	**German Translation**	**Cognate Status**	**Target NLD**
blokje	Block	C	0.83	1.69	6	bal	Ball	C	0.93
jas	Jacke	C	0.7	48.11	3	jurk	Kleid	NC	0.57
sok	Socke	C	0.88	3.11	3	schoen	Schuh	C	0.73
buik	Bauch	C	0.77	26.09	3	voet	Fuß	C	0.87
hoed	Hut	C	1.00	35.95	3	muts	Mütze	C	0.76
boom	Baum	C	0.87	52.25	3	wolk	Wolke	C	0.86
zon	Sonne	C	0.88	68.67	3	maan	Mond	C	0.76
balkon	Balkon	C	0.9	6.63	6	kelder	Keller	C	0.92
papegaai	Papagei	C	0.77	3.29	7	uil	Eule	C	0.8
koe	Kuh	C	1.00	18.55	2	varken	Schwein	NC	0.62
boot	Boot	C	1.00	95.93	3	trein	Zug	NC	0.67
deur	Tür	C	0.93	247.48	3	kamer	Zimmer	NC	0.8
fles	Flasche	C	0.84	45.71	4	doos	Karton	NC	0.62
krijtje	Kreide	C	0.9	0.59	5	potlood	Bleistift	NC	0.66
dokter	Doktor	C	0.98	244.07	6	verpleegster	Krankenschwester	NC	0.71
stofzuiger	Staubsauger	C	0.82	2.45	9	dweil	Mopp	NC	0.54
mier	Ameise	NC	0.72	2.54	3	spin	Spinne	C	0.88
druif	Traube	NC	0.76	1.35	4	meloen	Melone	C	0.84
stoep	Bürgersteig	NC	0.59	7.68	4	straat	Straße	C	0.83
tand	Zahn	NC	0.78	8.07	4	lip	Lippe	C	0.88
bord	Teller	NC	0.66	27.3	4	schaal	Schale	C	0.73
kikker	Frosch	NC	0.63	8.23	5	slang	Schlange	C	0.84
mandje	Korb	NC	0.63	2.2	6	emmer	Eimer	C	0.93
winkel	Geschäft	NC	0.51	65.13	6	bakker	Bäcker	C	0.94
gootsteen	Spülbecken	NC	0.65	2.81	7	keuken	Küche	C	0.85
komkommer	Gurke	NC	0.74	1.33	8	erwtje	Erbse	C	0.88
beschuit	Zwieback	NC	0.51	-	6	rozijntje	Rosine	C	0.85
juf	Lehrerin	NC	0.6	4.71	3	zus	Schwester	NC	0.64
tuin	Garten	NC	0.71	36.66	3	zandbak	Sandkasten	NC	0.74
broek	Hose	NC	0.42	67.28	4	riem	Gürtel	NC	0.6
glijbaan	Rutsche	NC	0.58	0.5	6	schommel	Schaukel	NC	0.73
brommer	Moped	NC	0.78	1.07	6	fiets	Fahrrad	NC	0.67
**German** **Prime**	**Dutch Translation**	**Cognate Status**	**Prime NLD**	**Word Frequency**	**Word Length**	**German** **Target**	**Dutch Translation**	**Cognate Status**	**Target NLD**
Saft	sap	C	0.75	11.58	4	Apfel	appel	C	0.9
Schiff	schip	C	0.75	169.1	3	Auto	auto	C	0.97
Messer	mes	C	0.8	48.19	4	Löffel	lepel	C	0.86
Fuß	voet	C	0.87	62.29	3	Bein	been	C	0.87
Hase	haas	C	0.88	7.21	4	Eichhörnchen	eekhoorntje	C	0.73
Pferd	paard	C	0.88	45.91	4	Reh	ree	C	1
Nilpferd	nijlpaard	C	0.9	0.94	7	Bär	beer	C	0.93
Ei	ei	C	0.9	21.81	1	Brötchen	broodje	C	0.87
Junge	jongen	C	0.93	326.98	4	Mädchen	meisje	C	0.78
Ohr	oor	C	1.00	27.56	2	Mund	mond	C	0.93
Flugzeug	vliegtuig	C	0.83	78.19	7	Hubschrauber	helikopter	NC	0.71
Sessel	zetel	C	0.86	4.88	5	Fenster	raam	NC	0.6
Buch	boek	C	0.87	106.3	3	Stift	potlood	NC	0.6
Stein	steen	C	0.88	44.88	4	Teich	vijver	NC	0.62
Schneemann	sneeuwman	C	0.89	0.98	6	Garten	tuin	NC	0.71
Strand	strand	C	0.95	39.02	6	Wald	bos	NC	0.6
Doktor	dokter	C	0.98	100.28	6	Feuerwehrmann	brandweerman	NC	0.77
Schal	sjaal	C	1.00	3.78	3	Lätzchen	slabbetje	NC	0.69
Zwieback	beschuit	NC	0.51	-	6	Pfannkuchen	pannekoek	C	0.71
Onkel	oom	NC	0.53	136.78	5	Bruder	broer	C	0.83
Mantel	jas	NC	0.55	22.21	6	Schuh	schoen	C	0.73
Nachtisch	toetje	NC	0.59	7.21	6	Müsli	muesli	C	1.00
Kino	bioscoop	NC	0.64	34.1	4	Supermarkt	supermarkt	C	0.96
Tisch	tafel	NC	0.68	96.62	3	Stuhl	stoel	C	0.98
Tablett	dienblad	NC	0.79	3.03	6	Deckel	deksel	C	0.92
Keks	koekje	NC	0.79	6.38	4	Brot	brood	C	1.00
Zimmer	kamer	NC	0.8	204.34	4	Bett	bed	C	1
Schlafanzug	pyjama	NC	0.58	2.36	9	Kleid	jurk	NC	0.57
Huhn	kip	NC	0.62	23.31	3	Schwein	varken	NC	0.62
Teller	bord	NC	0.66	13.23	4	Topf	pan	NC	0.69

(Note: word frequency is calculated by SUBLEX. “C” and “NC” in the cognate status represent “Cognate” and “Non-cognate”).
